# Long-Chain Bio-Based Nylon 514 Salt: Crystal Structure, Phase Transformation, and Polymerization

**DOI:** 10.3390/polym16040480

**Published:** 2024-02-08

**Authors:** Zihan Li, Lei Zhang, Xiaohan Zhang, Tianpeng Chen, Pengpeng Yang, Yong Chen, Huajie Lin, Wei Zhuang, Jinglan Wu, Hanjie Ying

**Affiliations:** 1 National Engineering Technique Research Center for Biotechnology, State Key Laboratory of Materials-Oriented Chemical Engineering, College of Biotechnology and Pharmaceutical Engineering, Nanjing Tech University, No. 30, Puzhu South Road, Nanjing 211816, China; 15895877898@163.com (Z.L.); zhang_xh31@163.com (X.Z.); yang-pengpeng@njtech.edu.cn (P.Y.); chenyong1982@njtech.edu.cn (Y.C.); weizhuang@njtech.edu.cn (W.Z.); wujinglan@njtech.edu.cn (J.W.); yinghanjie@njtech.edu.cn (H.Y.); 2 Nanjing Biotogether Co., Ltd., No. 8, Shuangfeng Road, Nanjing 211806, China; zhanglei2301@163.com; 3 SINOPEC Ningbo Research Institute of New Materials, No. 88, Mianfeng Road, Ningbo 315200, China

**Keywords:** 1,5-pentanediamine-tetradecanedioate, long-chain bio-based nylon 514 monomer, crystal structure, phase transformation, nylon polymerization

## Abstract

Nylon 514 is one of the new long-chain bio-based nylon materials; its raw material, 1,5-pentanediamine (PDA), is prepared by biological techniques, using biomass as the raw material. The high-performance monomer of nylon 514, 1,5-pentanediamine-tetradecanedioate (PDA-TDA) salt, was obtained through efficient crystallization methods. Here, two crystal forms of PDA-TDA, anhydrous and dihydrate, were identified and studied in this paper. From the characterization data, their crystal structures and thermal behaviors were investigated. Lattice energy was calculated to gain further insight into the relationship between thermal stability and crystal structures. The contribution of hydrogen bonds and other intermolecular interactions to the crystal structure stability have been quantified according to detailed Hirshfeld and IRI analyses. Additionally, the transformation mechanism of the anhydrate and dihydrate was established through a series of well-designed stability experiments, in which the temperature and water activity play a significant role in the structural stability of crystalline forms. Eventually, we obtained nylon 514 products with good thermal stability and low absorption using stable dihydrate powders as monomers. The properties of nylon 514 products prepared by different polymerization methods were also compared.

## 1. Introduction

Nylons, an industrial polymer containing recurring units—[NH-CO]—on the molecular backbone, scientifically known as Polyamide (PA), were first introduced in the 1940s for use in stockings [1,2]. Due to their excellent mechanical attributes, stable chemical properties, superior heat resistance, and electrical insulation features [3,4,5], nylons are among the most widely employed synthetic fibers used in textiles [6]. Currently, long carbon chain nylon is widely used in important fields such as the manufacture of machinery, automobiles, electronic appliances, and aerospace items due to its low water absorption, strong dimensional stability, wear resistance, and other excellent advantages. The common long-chain nylons on the market today mainly include nylon 11, nylon 12, nylon 1212, nylon 1313, etc. [7,8]. The existing varieties of and production methods for these nylons are currently not sufficient to supply the demand of the domestic market, so it mainly relies on imports. Meanwhile, green processes are considered in industries, particularly in recent years, due to the increasing environmental demands from governments and customers [9]. Research on bio-based nylons is considerably significant in the interdisciplinary fields of chemical engineering, biology, and materials [10]. It is well known that the extraction and production methods of conventional nylon raw materials are still dominated by traditional fossil energy sources [11], and these processes involve toxic chemical substances, causing serious pollution to the environment and affecting the quality of human life [12,13]. Furthermore, nylon 6 and nylon 66, which account for 90% of the total value of the nylon market, also have relatively high water absorption (1.8% vs. 3.5%) [14]. Hence, it is of great practical significance to develop bio-based long-chain nylon based on renewable resources [15], which leads the trending direction regarding the development of nylon in terms of its consistency with the sustainable development concept [16,17].

**Figure 1:**
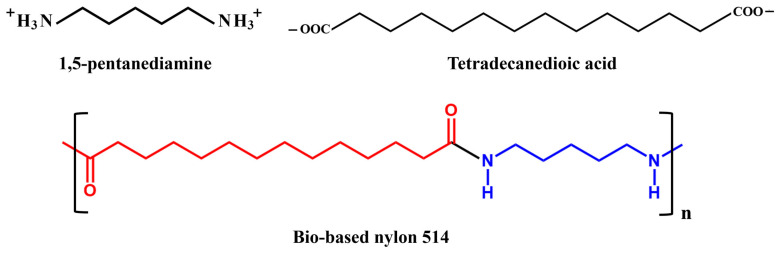
Molecular structure of PDA–TDA and bio-based nylon 514.

1,5-pentanediamine (PDA) is the main raw material for the production of series bio-nylon 5X, which opens up a new green production method due to its valuable industrial applications [18,19,20]. With the increasing maturity of biotechnology for preparing PDA from biomass resources such as corn straw [21,22,23], the synthetic routes and small-scale industrial application of series nylon 5X have been preliminarily established [24]. Nylon 514, which is polymerized by the crystalline salts of 1,5-pentanediamine-tetradecanedioate (abbreviated to as PDA-TDA; see Figure 1, C_19_H_40_N_2_O_4_, M.W. 360.53 g/mol). Considering that the quality of monomers is vital to the polymerization process of the nylon products targeted foe their excellent performance, the crystallization technology was chosen to prepare and purify the monomers [25,26]. Interestingly, two crystalline forms of PDA-TDA were obtained during the crystallization, an anhydrous form and a dihydrate form. This observation prompted us to inquire into the differences between the crystal structures and physicochemical properties of the two forms.

As seen in Figure 1, hydrate–anhydrate phase transformations are complicated processes. Defining the environmental conditions to ensure that the material stays within an acceptable range is useful to minimize the risk of phase conversion during manufacturing or storage [27,28]. Moreover, the anhydrous forms and hydrates of a compound may sometimes differ greatly in terms of bulk density, hygroscopic properties, stability, and mechanical properties [29,30,31,32,33,34,35]. Intermolecular interactions analysis, thermodynamic analysis, and quantitative calculation as promising and useful tools for polymorph exploration are commonly used to assess the stability and the structure–activity relationship of target crystalline forms, as well as to gain insight into the mechanism of transformation between them [36,37,38,39,40]. Meanwhile, the solid-state transformations (SST) [41,42,43] and solution-mediated polymorphic transformations (SMPT) [44,45,46,47] experiments were usually designed to investigate the transformation mechanism between the crystal forms in recent research.

In this study, the crystal structures and thermodynamic behaviors of PDA-TDA anhydrate and dihydrate were characterized using qualitative analytical methods. The stability of the structures of the two forms was analyzed in detail, in terms of their intermolecular interactions and spatial arrangement, using Hirshfeld surface analysis, IRI analysis, and lattice energy calculations. Furthermore, we explored the effect of heat and humidity on the polymorphic outcome through a series of transformation experiments to gain a more particular knowledge of the phase transition process. Finally, nylon 514 was polymerized using dihydrate crystals, which was the most stable crystal form of PDA-TDA, as the raw material. The thermal properties, water absorption, and molecular weight of the obtained nylon 514 products were also further investigated.

## 2. Materials and Methods

### 2.1. Materials

1,5-pentanediamine (PDA, 99.5% purity, B.P. 180 °C, M.W. 102.18 g/mol) was prepared in our laboratory using the whole-cell catalysis method of L-lysine decarboxylation. Tetradecanedioic acid (TDA, 99.0% purity, M.P. 124 °C, M.W. 258.35 g/mol) was purchased from Shanghai Macklin Biochemical Co., Ltd. (Shanghai, China). Deionized water was obtained from an ultrapure water system form Nanjing YPYD technology development Co., Ltd. (Nanjing, China).

### 2.2. Preparation of the Crystal Forms

Both the anhydrous and dihydrate forms of PDA-TDA were prepared by anti-solvent crystallization. The difference is that the solvent is methanol, and the anti-solvent is 2-propanol (1:7 *v*/*v*) when anhydrate is prepared, while the solvent for preparing dihydrate is aqueous solution, and the anti-solvent is 2-propanol (1:9 *v*/*v*). The specific crystallization experiments for the obtained PDA-TDA forms and the cultivation procedures of single crystals are listed in the Supplementary Materials. After the experiment, the obtained PDA-TDA crystals were filtered, washed, and dried. The structures of two crystalline products were identified by PXRD for consistency.

### 2.3. Single Crystal X-ray Diffraction (SCXRD)

Preliminary examination was performed, and crystallographic data of the two crystalline powders were collected on a Bruker SMART APEX-II CCD diffractometer with graphite-monochromated Mo Kα radiation (λ = 0.71073 Å) at room temperature. Data collection and reduction were accomplished using the SAINT [48] program through the Bruker APEX3 2018 software. The unit cell parameters and the orientation matrix for data collection were obtained through least-squares methods on the squared structure factors F^2^ [49]. The initial positions of all non-hydrogen atoms were obtained through direct methods of the SHELXS-97 program, and the structure was solved and refined using SHELXL-2018/3 [50] through the graphical user interface XSeed [51]. Measurement details of refinements processed using PLATON [52], MERCURY 3.3 [53], and Olex2 1.3 [54] were used for structure visualization and the acquisition of standard diffraction patterns. Crystallographic data for anhydrate and dihydrate were obtained in CIF format and have been deposited into the Cambridge Structural Database, acquiring the CCDC numbers 2112957 and 2112969.

### 2.4. Spectroscopy Analysis

Powder X-ray diffraction (PXRD) patterns were collected on a Rigaku SmartLab diffractometer system (Shanghai Bright Industrial Co., Ltd. Shanghai, China) equipped with Cu Ka radiation (λ = 1.5418 Å, 40 kV, 40 mA). Samples were scanned over the range 5° ≤ 2θ ≤ 40° in continuous scan mode, with a rate of 10° min^−1^ and a step size of 0.02°. The infrared (IR) spectroscopy was carried out by a Nicolet iS5 FT-IR spectrometer with an ATR reflectance attachment for solid powders. Each spectrum was recorded over the range of 4000–400 cm^−1^ with a resolution of 4 cm^−1^ at room temperature.

### 2.5. Thermal Analysis

Thermal gravimetric analysis and differential scanning calorimetry (TGA-DSC) measurements were performed using a METTLER TGA/DSC 1 instrument (METTLER TOLEDO Technology (China) Co., Ltd. Shanghai, China). Approximately 3–5 mg samples were weighed in an alumina pan (40 μL) and scanned in a 30 to 300 °C temperature range at a 10 °C/min heating rate under a nitrogen atmosphere (flow rate 40 mL/min). The temperature of the initiation of melting and decomposition for the samples was specified in the DSC and TGA curves, respectively. The enthalpy of the results was given in the unit J/g.

### 2.6. Hirshfeld Surface Analysis (HSs)

The Hirshfeld surface, 2D-fingerprint plots, and the energy frameworks were generated using CrystalExplorer 17.5 software [55,56,57], according to the results of SCXRD analysis in this work, to explore intermolecular interactions as well as to quantify the intermolecular contacts on each of the forms [58,59]. The normalized contact distance (*d*_norm_) surface was used for identifying and quantifying intermolecular interactions that are relevant in the crystal lattice. Molecular wave functions were obtained using the built-in Tonto utility at an “accurate” setting using the B3LYP/6-31G(d,p) level of theory. The monomer wavefunction was used to obtain accurate values using Grimme’s D2 dispersion correction [60,61].

### 2.7. Computational Details

IRI (interaction region indicator) analysis is a visualization index based on electron density and its derivatives [62]. IRI isosurface analysis can be used to visualize the non-covalent interactions and weak interactions between the molecular crystals. Gaussian 09 was used to perform the geometry optimization and energy calculation at the B3LYP/6-311G(d,p) level, with a 6-21G basis set [63,64,65], through format conversion; then, the wave function file was obtained. Multiwfn 3.8 [66,67] was used to provide cube-format cube files and identify the isosurface. All isosurface maps were visualized and rendered using VMD 1.9.3 [68]. The lattice energy of the two forms of PDA-TDA was calculated using the Materials Studio 7.0 DMol^3^ module [69]. First, the experimental crystal structures were optimized with the unit cell parameters fixed, then all calculations were performed using the generalized gradient approximation (GGA) by Perdew–Burke–Ernzerhof (PBE) [70] as the exchange-correlation density functional, with the addition of Grimme’s D2 dispersion correction, and DNP was chosen as the basis set before the calculations.

### 2.8. Stability Experiments

The stability of anhydrate and dihydrate was illustrated under different thermal and humidity conditions. Approximately 2 g of anhydrate and dihydrate powders were placed into several dishes, which were then stored at room temperature (R.T.), 40 °C, 60 °C, 80 °C, and 90 °C for one week, as well as at 110 °C for 15 h, respectively. A small number of samples were removed every day for PXRD characterization. Relative humidity (RH) was another important factor to be studied. A total of 400 mg of two forms of crystalline products, respectively, were weighed out and placed in Petri dishes, under varying relative humidity [71]. Samples were also removed for PXRD evaluation after four months. Additionally, seven organic solvents (ethanol, 1-propanol, 2-propanol, 1-butanol, ethyl acetate, DMF, and DMSO) were used for phase transition. A small amount of excess anhydrous and dihydrate solid powder was weighed, and then 8 mL of the above organic solvent was added to each respectively, and the mixture was stirred at 500 r/min for one week to reach a solid–liquid two-phase equilibrium. After the test period, the turbid solution was filtered to obtain the residual solid, which was then dried, and the solid powders were identified by PXRD.

### 2.9. Polymerization Experiments

Bio-nylon 514 was prepared using two polymerization methods, melting polymerization (MP) and direct solid-state polymerization (DSSP), which were used to evaluate the performance of the nylon products obtained by the different methods. The obtained polymers can be characterized and analyzed after being completely dried in a vacuum oven. The specific polymerization procedures, as well as the characterization and analysis techniques of the obtained nylon 514 polymers, are listed in the Supplementary Materials.

## 3. Results and Discussion

### 3.1. PXRD Analysis

**Figure 2:**
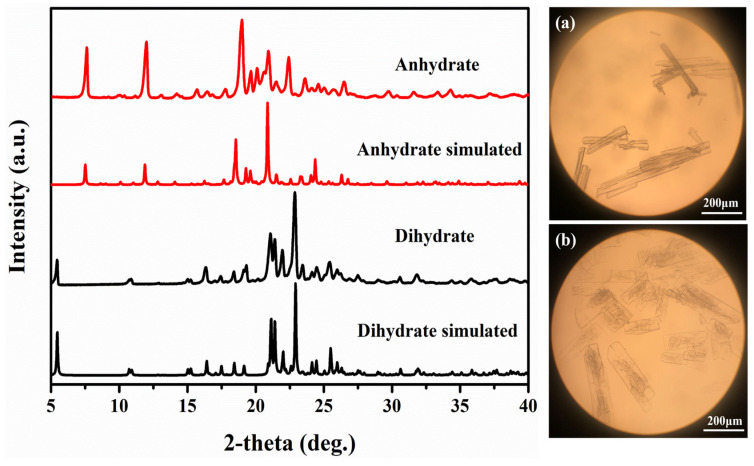
PXRD patterns of PDA–TDA (**left**) and images of the crystalline products of PDA–TDA (**right**): (**a**) anhydrate; (**b**) dihydrate. Both views are obtained at 16 × 4 magnification.

The PXRD patterns of the two crystalline phases of PDA–TDA are identified and shown in Figure 2. The fingerprint can be easily distinguished owing to their respective characteristic peaks (2θ = 8.49°, 12.72°, 19.22°, and 21.45° for the anhydrate, and 2θ = 5.47°, 21.17°, 21.44°, and 22.94° for the dihydrate). The PXRD measurements confirmed that all the samples exhibited pure phases, similar to their single crystals. The two forms are also easily distinguished by their different crystal habits; the anhydrous form is thin and stick-like, while the dihydrate is flake-like.

### 3.2. FTIR Spectroscopy Analysis

**Figure 3:**
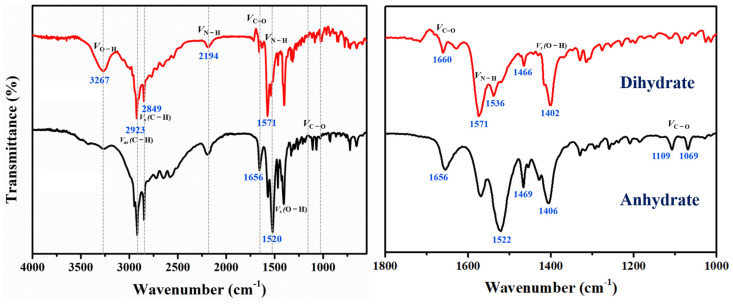
FTIR spectroscopy patterns of PDA–TDA salts; the black line is anhydrate, while the red line is dihydrate.

Figure 3 shows the IR spectrums of the anhydrate and dihydrate markedly different spectral characteristic bands [72]. The band at 3267 cm^−1^ of dihydrate, which is typically caused by the -OH stretching vibration of water molecules, differs from the behavior of the anhydrous phase [73]. The band at ca. 2200 cm^−1^ is assigned to the stretching vibration of the ionized amine groups (NH_3_^+^), which is the most common characteristic peak of nylon salts.

Many legible differences can be seen in the fingerprint area at 1700–1400 cm^−1^, such as the characteristic bands at 1660, 1571, and 1402 cm^−1^ of dihydrate, and at 1656, 1522, and 1406 cm^−1^ of anhydrate. Among them, the mentioned bands at ca. 1660 cm^−1^ and 1410 cm^−1^ are the antisymmetric stretching vibration of NH_3_^+^ and the symmetric stretching vibration of COO^−^, respectively [74]. In addition, the absorbing bands in the range of 1200–1000 cm^−1^ were the result of the H-bonding interactions between the N–H of PDA and the C=O of TDA.

### 3.3. Crystal Structures Analysis of PDA-TDA Anhydrate and Dihydrate

Crystallographic data for anhydrate and dihydrate is summarized in Table 1. There are significant differences between the two crystalline forms in terms of their crystal constants, density, volume, etc. The hydrogen bond lengths and angles are also given in Table S1.

To get an insight into the crystal packing and interactions of the two crystalline forms of PDA–TDA, the crystal structures, intermolecular interactions, and packing modes were deeply analyzed.

**Figure 4:**
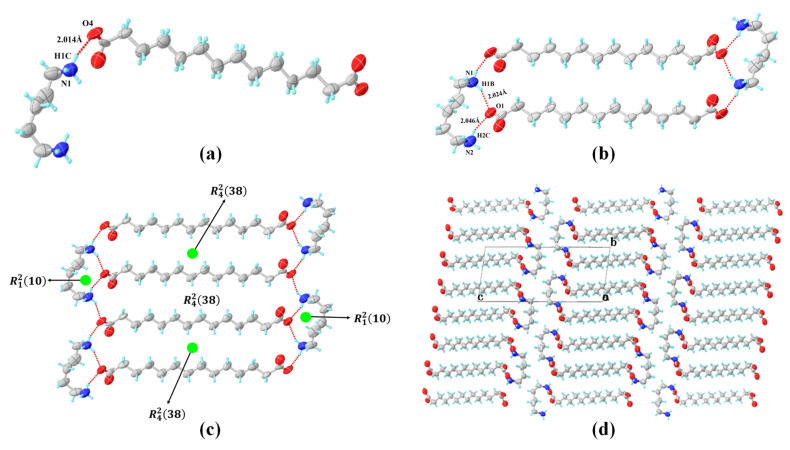
(**a**) The smallest asymmetric unit cell with the selected atom labeling scheme of anhydrate. The ellipsoids are shown at a 50% probability. (**b**) The structure of double layer unit of the anhydrate. (**c**) A view of the packing motifs of anhydrate, with hydrogen supramolecular synthons between PDA-TDA. (**d**) The 3D supramolecular framework of the anhydrate, with an open 1D channel along the *c* axis.

Anhydrate: The anhydrate of PDA–TDA was a colorless, stick-like crystal. The asymmetric unit cell only consists of one molecule each of PDA and TDA, which crystallized in the triclinic crystal system and *P*-1 space group. The asymmetric unit, packing motifs, and 3D supramolecular framework of the anhydrate are illustrated in Figure 4. The hydrogen bonding N1–H1C···O4 (2.014 Å, Figure 4a) in the asymmetric unit cell of anhydrate indicates strong close contacts between the PDA and TDA molecules. Figure 4b shows a double layer composed of asymmetric units in which the amino functional group of the PDA cation can interact with two carboxyl groups of the TDA anions via hydrogen bonding. Notably, due to the bending deformation of the carbon chain skeleton of the PDA molecule, the distance between the two amino groups is significantly shortened. Then, an intramolecular interaction is formed simultaneously according to the hydrogen bonds between the two nitrogen atoms on the amino group of PDA and the one oxygen atom on the carboxyl group of TDA (N1–H1B∙∙∙O1, 2.024 Å; N2–H2C∙∙∙O1, 2.046 Å). In Figure 4c, it can be seen that, based on the hydrogen bonds between the adjacent double layers, in parallel, three $R_{4}^{2}\left(38\right)$ and four $R_{1}^{2}\left(10\right)$ supramolecular synthons are established. Additionally, Figure 4d depicts a 3D supramolecular framework of the anhydrous form further assembled by the 2D supramolecular layer along the *c* axis (face 100), where the PDA molecules are stacked in an alternately anti-parallel arrangement.

**Figure 5:**
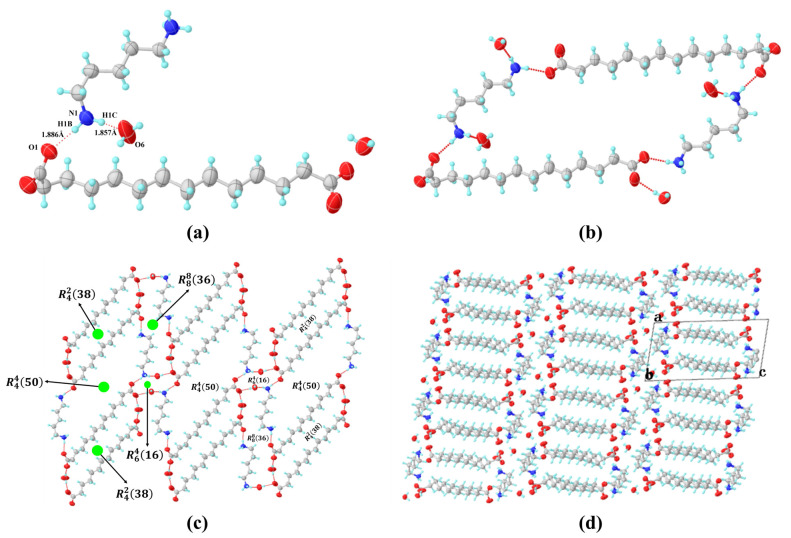
(**a**) The smallest asymmetric unit cell with the selected atom labeling scheme of dihydrate. The ellipsoids are shown at a 50% probability. (**b**) The structure of double layer unit of dihydrate. (**c**) A view of the stacking structure of dihydrate, with supramolecular hydrogen synthons between the interactions of water and PDA-TDA. (**d**) The 3D supramolecular framework of the dihydrate, with an open 1D channel along the *c* axis.

Dihydrate: The shape of the dihydrate of PDA-TDA crystallizes with a flake-like structure, and the crystal system and space group are consistence with the anhydrous form. Figure 5 shows the intermolecular interactions, hydrogen bonding synthons, and packing 3D networks of dihydrate. In Figure 5a, a close contact N1–H1B···O1 (1.886 Å) in the asymmetric unit indicates strong hydrogen bonding between the PDA and TDA molecules. Two water molecules are present around the host PDA and TDA ions: one is through N1-H1C∙∙∙O6 contact (1.857 Å) resulting from the amino group of PDA cation, and the other occurs around the carboxyl group of the TDA anion, but is not connected to it due to a slightly longer spatial distance. The homodimer, as the basic building block (see Figure 5b), is composed of two asymmetric units of dihydrate and is further linked as a bimolecular chain via N–H···O bonds. Figure 5c shows the packing view of these homodimers, which are further extended through hydrogen bonding synthons, creating the 2D structure. The two host molecules formed the largest $R_{4}^{4}\left(50\right)$ hydrogen bonding synthons. Meanwhile, two $R_{4}^{2}\left(38\right)$ and one $R_{8}^{8}\left(36\right)\;$supramolecular graph sets involving the two lattice water molecules have also been formed through the connection between the host and water molecules. Additionally, an $R_{4}^{4}\left(16\right)$ synthon consisting of the two host water molecules and the amino groups of PDA and the carboxyl groups of TDA via the N–H···O and O–H···O interactions, bridged well two neighboring hydrogen synthons units in the *bc* plane. Furthermore, Figure 5d depicts a complete 3D packing network of dihydrate along the *c* axis, based on the abundant intermolecular hydrogen synthons (face 010), thereby forming a more densely packed network.

Table 2 shows the torsion angle comparison of the two PDA-TDA crystalline forms. Among them, the two dihedral angles O1-C1-C2-C3 and O2-C1-C2-C3 are formed by two oxygen atoms on the carboxyl group of TDA and its carbon chain backbone, respectively. The difference is mainly due to the difference in intermolecular interactions and stacking modes, which are attributed to the participation of water molecules in dihydrate. Specifically, a comparison of torsion angles in the asymmetric units of the anhydrate and dihydrate is illustrated in Figure S1.

The water molecules arranged in the lattice will also have a great influence on the stability of the crystal. Figure S2 shows the comparison of crystal density (1.068 g/cm^3^ for anhydrate and 1.134 g/cm^3^ for dihydrate) and packing efficiency (0.643 for anhydrate and 0.683 for dihydrate) between the two phases. According to the density rule [75], the crystal form with the higher density is consistent with better crystal packing and higher stability. Consequently, the dihydrate crystal of PDA-TDA may have a higher stability than the anhydrous form. To more intuitively describe the correlation between the structure and stability of the two forms, further theoretical analysis of the intermolecular interactions was performed using the Hirshfeld surface analysis [56].

### 3.4. Hirshfeld Surface Analysis

**Figure 6:**
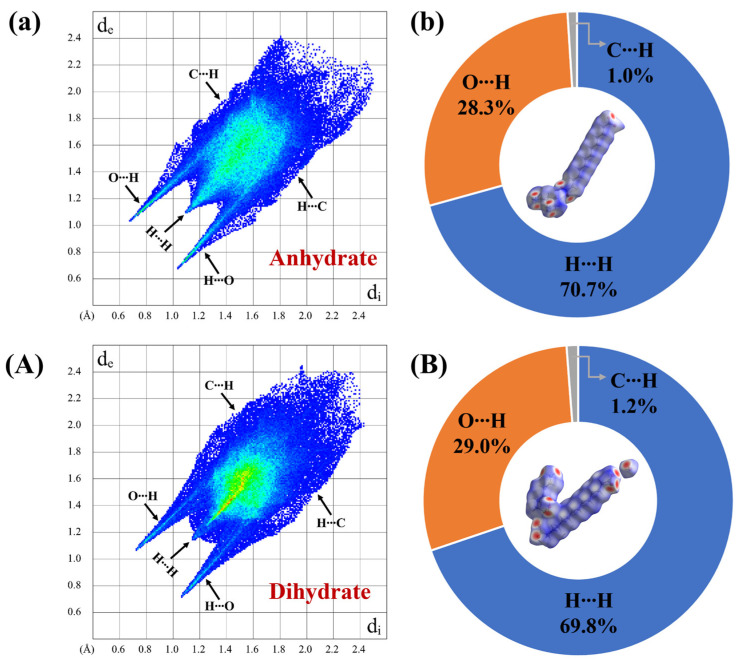
The 2D Fingerprint plots showing the close contacts in the anhydrate (**a**) and dihydrate forms (**A**). (**b**,**B**) The fan chart elucidates the percentage contributions to the calculated Hirshfeld surfaces of the two forms. The HS surface mapped over *d_norm_* is in the color range of −0.6204 to 1.6275 a.u./−0.6979 to 1.2588 a.u., respectively.

The Hirshfeld surface analysis (HSs) was used to quantify the molecular packing and contributions of the intermolecular interactions present in the crystal structures of PDA-TDA anhydrate and dihydrate [76]. As can be seen from Figure 6, the HSs maps of the two PDA-TDA salts graphically indicate the closer hydrogen bond contacts and their corresponding fingerprints. Figure 6a,A shows the five similar intermolecular interactions present in the two crystalline forms. Then, Figure 6b,B elucidates the percentage contribution of these close contacts, as we were interested in comparing their strengths. The hydrogen bond skeleton H···H contacts in the anhydrate and dihydrate accounted for the largest percentage of total interactions (70.7% vs. 69.8%, respectively). In addition, the O···H/H···O contacts comprise the majority of the interactions (28.3% for anhydrate and 29.0% for dihydrate), and the excess interaction in dihydrate should be caused by the hydrogen bond generated by the participation of water molecules. Correspondingly, even the C···H/H···C interactions were observed to make a small contribution in proportion (only 1.0% for anhydrate, while 1.2% for dihydrate); it is also an important contact and varies with the specific crystal structure of the multicomponents. The percentage contribution of the anhydrous form and dihydrate components are extremely similar overall, suggesting that the dihydrate may exhibit higher stability than the anhydrous form, considering the major contribution of the hydrogen bond interactions. The conjectural conclusion is consistent with the results of the crystal density comparison above.

Additionally, a more detailed description of the HSs in the two salts is plotted in Figures S3 and S4 for clarity. In particular, the specific C–H···O van der Waals interactions in the anhydrous phase and the dihydrate are provided in Figure S5.

### 3.5. Computational Analysis

**Figure 7:**
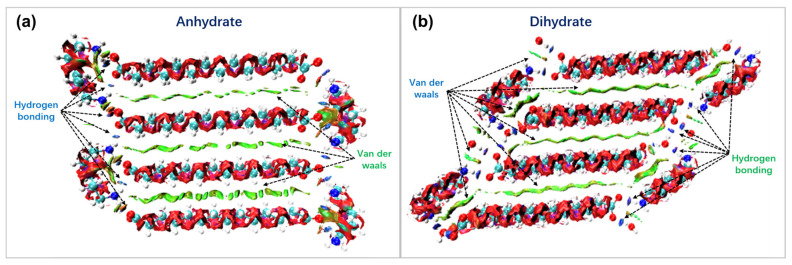
IRI plot isosurface of the (**a**) anhydrate and (**b**) dihydrate structures of PDA-TDA.

IRI analysis was carried out on the crystal structures of the two phases of PDA-TDA to get an insight into the intermolecular interactions. It should be noted that the bluer the isosurface color, the stronger the hydrogen bonding. The green area on the isosurface represents the weak van der Waals interactions, while the red area indicates the steric effect in the ring or cage structures. The redder the color, the stronger the steric hindrance. Similar IRI plots of the anhydrate and dihydrate forms elucidated in Figure 7 are consistent with the percentage contribution of the 2D fingerprint analysis above. As noted, the isosurfaces of the N–H∙∙∙O or O–H∙∙∙O hydrogen bonds are deep blue, while the van der Waals interactions are colored green. Among them, the dihydrate shows relatively stronger hydrogen bonds and van der Waals forces than those exhibited by the anhydrous form, owing to the presence of the extra O–H∙∙∙O hydrogen bond interaction. It is worth noting that both of the two crystal structures contain a large number of C–H∙∙∙O weak van der Waals interactions in the lattice, which can lead to crystal instability in general. However, due to the strong steric hindrance effects produced by the long carbon chain of the TDA molecules (abundant red hindrance effects on the TDA isosurfaces), the crystal exhibits extremely strong stability.

Moreover, the lattice energies of the two forms that can be used to evaluate stability are also calculated, using the software Materials Studio 7.0, and the calculated data are listed in Table S2. It was observed that the lattice energy of anhydrate is −169.30 kJ/mol, while the dihydrate shows a higher energy of −164.78 kJ/mol. Overall, based on the preceding evidence, it can be concluded that the N–H···O and O–H···O hydrogen bonds contribute greatly to maintaining the crystal structure of the dihydrate salt in terms of the calculation results of the HSs, IRI analysis, and lattice energy of the two crystalline forms.

### 3.6. Thermal Analysis

**Figure 8:**
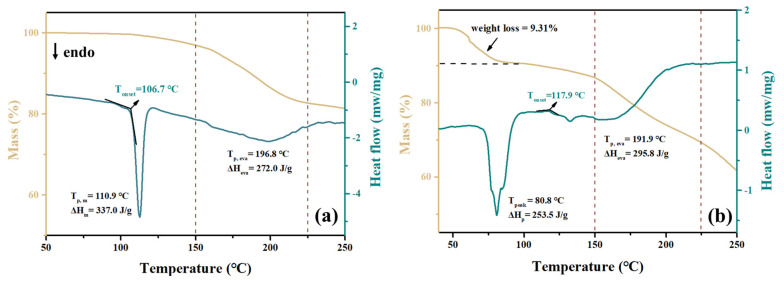
TGA and DSC curves of the anhydrate (**a**) and dihydrate (**b**) of PDA–TDA.

To further explore the thermal behaviors of the anhydrate and dihydrate salts, TGA and DSC measurements were performed. Apparently, the anhydrate of PDA-TDA shows no weight loss (Figure 8a) before its melting point, while the dihydrate has a weight loss of 9.31% (Figure 8b) at 80.8 °C in the DSC profiles. The endothermic peak in the DSC curve was attributed to the loss process of two crystal water molecules in the dihydrate lattice (theoretical: 9.08%). The anhydrous compound begins to show a sharp endotherm at 106.7 °C on the DSC curve, corresponding to its melting temperature. In contrast, there is only a wide and short peak (T_onset_: 117.9 °C) on the DSC thermogram of dihydrate after the endothermic peak of the water loss, and its TGA curve shows a simultaneous downward trend. This phenomenon indicates that the lattice of the dihydrate salt begins to collapse after the loss of water molecules, and then starts to melt.

In particular, the mass loss after the melt temperature observed for both salts in the temperature range between 150–225 °C (the area inside the two brown dotted lines) is not decomposition, but rather the polycondensation of water that is formed upon polymerization of the nylon salt [77], which occurs during the dynamic heating of the TGA/DSC measurement. This is further supported by the observed endotherms in the DSC curve, which correspond to the enthalpy of water evaporating under these conditions. The evaporation enthalpy of the water produced by nylon polymerization was 272.0 and 295.8 J/g respectively, which is close to the enthalpy of two water molecules from the heat of the dihydrate (253.5 J/g). In principle, the nylon salts show distinct endotherms of melting and reaction during the heat process. A melting endotherm corresponding to the melting of the salt crystal forms first and at a higher temperature, a second endotherm corresponds to the polymerization reaction, i.e., evaporation of the formed water. The observed difference between the determined enthalpies indicates that the dihydrate salt produces a slightly higher amount of polycondensation water, thus implying that the dihydrate of PDA-TDA is a better-balanced (with higher quality and equal end groups) salt compared to the anhydrous form.

Detailed thermodynamic information for the anhydrate and dihydrate is listed in Table 3. The results regarding melting points, enthalpy of evaporation, and lattice energy indicated the greater thermal stability of the dihydrate, which is consistent with the results of the previous quantitative calculation.

### 3.7. Stability Analysis

**Figure 9:**
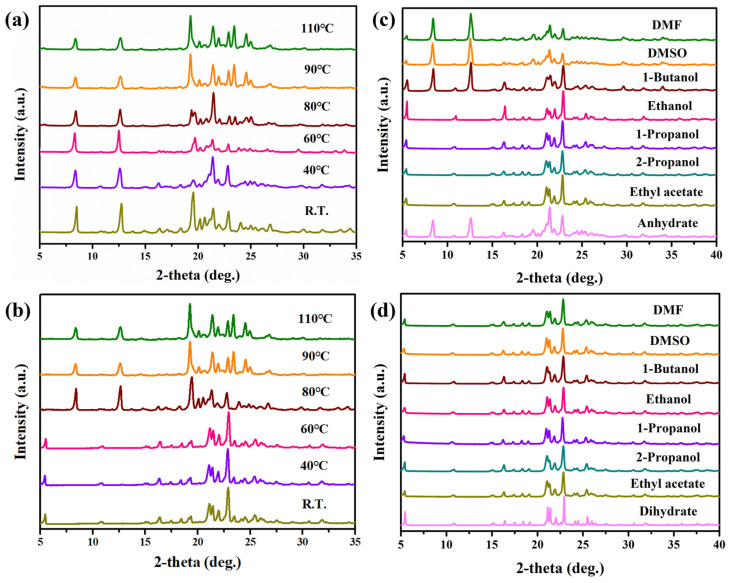
Results of SST and slurry experiments of the anhydrate (**a**–**c**) and dihydrate (**b**–**d**) under different temperature and solvent conditions, respectively.

The effects of temperature and solvents on the stability of the two forms of PDA-TDA were demonstrated by the SST and slurry experiments in Figure 9, evaluating the stability and the transition conditions of the crystals in different environments. Figure 9a,b depicts the thermal stability of the anhydrate and dihydrate at different temperatures, respectively. Upon heating, the anhydrous phase exhibits high thermostability, maintaining its structure even at 110 °C for 15 h (Figure 9a), while the dihydrate undergoes desolvation and converts to the anhydrate when exposed to 80 °C for 2 days (Figure 9b). Moreover, Figure 9c,d shows the slurry results in seven organic solvents. Contrary to the SST results, the dihydrate maintained itself well and displayed high stability in all solvents (Figure 9d). The anhydrate can retain its structure only in DMF, DMSO, and 1-butanol (Figure 9c), while converting to the dihydrate in the remaining four solvents. Stability analysis results also indicated that both the two crystalline forms can achieve mutual conversion under appropriate conditions. Moreover, we suggest that the thermal stability in the heat-induced transition may be dependent upon the intermolecular interaction and crystal packing structures.

The hygroscopicity experiments, with the samples subjected to various humidity levels for four months, were carried out, as recorded in Figure S6. Notably, it can be observed that the anhydrous phase presents a strong moisture absorption and can only maintain its structure when RH = 0%. Once this relative humidity is exceeded, it can be converted from anhydrous to dihydrate. By contrast, the dihydrate exhibits excellent stability at all relative humidity levels, except for RH = 0%, indicating strong stability at high hygroscopicity. In particular, the dihydrate can be partially transformed into anhydrate when the RH = 0%, and the PXRD pattern in this situation is the mixture of the two crystal forms.

### 3.8. Transitions between the Dihydrate and Anhydrate

**Scheme 1:**
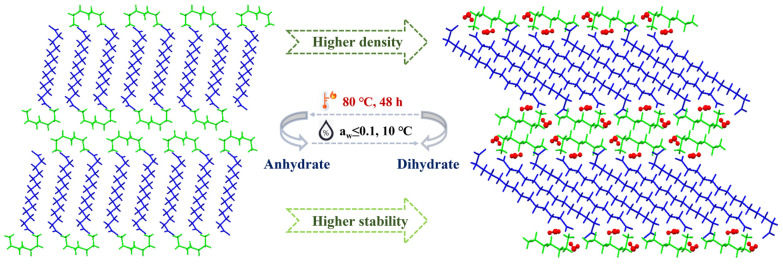
Interconversion pathways of the anhydrate and dihydrate forms of PDA-TDA.

According to the above thermal stability and hygroscopicity analysis, it can be concluded that the dihydrate is more stable than the anhydrous form of PDA-TDA. At the same time, the transformation process between the two crystalline forms is reversible under the appropriate conditions. To further shed light on this discovery, the slurry transformation experiments for PDA-TDA anhydrate and dihydrate in regards to the temperature and water activity, two factors were evaluated in a water–ethanol binary system. The mixture phase was fully stirred and held for 24 h to achieve full phase equilibration at the beginning, and then the dependence of the two powders on water activity (a_w_) in the ethanol–water mixtures was measured at different temperatures. The PXRD characterization results are evaluated in Figure S7. We can see that even if the experimental conditions are strictly controlled at 10 °C and a_w_ = 0.1, the anhydrous form of PDA-TDA is still transformed into a dihydrate. This result illustrated that the hydrate zone was rather wider than the anhydrous zone of PDA-TDA. Finally, Scheme 1 shows the transformation relationship between the PDA-TDA anhydrate and dihydrate, taking into account the conclusions of the above stability and slurry experiments.

### 3.9. Polymerization Experiments Analysis

**Figure 10:**
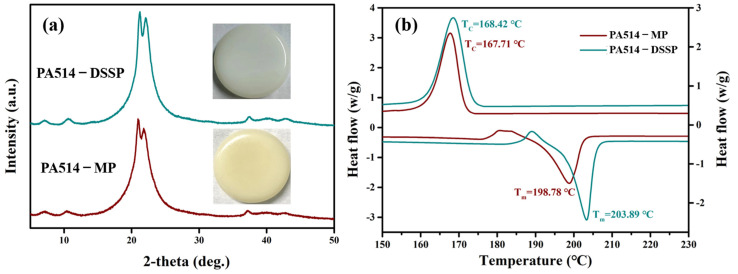
(**a**) Morphology and the corresponding PXRD patterns of the PA514 products. (**b**) Thermodynamic analysis of the PA514 products using MP and DSSP methods, respectively.

Polymerization experiments were performed on nylon 514 using melting polymerization (MP) and direct solid-state polymerization methods (DSSP), using the dihydrate powders as raw materials. Based on the qualitative and thermodynamic analyses of the obtained products, the performance was further compared, as illustrated in Figure 10 and Figure S8. As noted, Figure 10a displays the almost identical PXRD patterns of the PA514 nylons, according to the MP and DSSP polymerization methods, indicating that the obtained nylon polymers are the same. The FTIR analysis shown in Figure S8 also proves this result. Moreover, the color of the products, according to DSSP polymerization, is lighter than that of MP, and the characteristic peak intensity of the former is also slightly higher than that of the latter, indicating that the effect of DSSP polymerization is relatively better.

Meanwhile, the thermodynamic analysis of the PA514 nylons was also carried out, as elucidated in Figure 10b. Notably, the polymer prepared using DSSP shows higher thermal stability in terms of its melting temperature (T_m_, 203.9 °C vs. 198.8 °C) and crystallization temperature (T_C_, 168.4 °C vs. 167.7 °C). Besides, small rise melting peaks can be seen before the melting points of nylons, which are caused by inconsistencies in the crystallinity of the polymer. The regions with low crystallinity will melt first and recrystallize rapidly, and then melt together with the regions with high crystallinity. Given that the melting points of PA514 nylons obtained by two different polymerization methods are about 200 °C, it can be seen that PA514 is a new type of high-temperature resistant nylon.

To further investigate the properties of the obtained PA514 polymers, the molecular weight, intrinsic viscosity, and water absorption of the products were tested, and the results are summarized in Table 4. Obviously, both the *M*_n_ and *M*_w_ of PA514 polymerized by DSSP are higher than the MP. In addition, the former has a lower PDI than the latter. Therefore, the above GPC results suggest that the DSSP method exhibits better polymerization performance, and the molecular weight distribution of the obtained product is more concentrated. It is well known that the higher the molecular weight of the polymer, the higher the viscosity. The intrinsic viscosity data for the PA514 products were in agreement with the results of molecular weight. Furthermore, both the PA514 polymers prepared by the DSSP and MP method showed excellent low water absorption, especially the nylon polymerized by DSSP, which is a feature that is not possessed by many short-chain nylons on the market. According to the above observation and analysis, it can be concluded that PA514 is a new type of bio-based long chain nylon, with practical industrial application prospects.

## 4. Conclusions

In summary, we demonstrate that PDA-TDA exists in two forms, an anhydrous form and a dihydrate, which can be switched by performing the transformation experiments. The structures, characterization, and thermodynamic properties were investigated using several analytical tools. In addition, the difference in the packing conformation and intermolecular interactions between the anhydrous form and the dihydrate was fully elucidated using the Hirshfeld surface analysis, IRI analysis, and lattice energy, illustrating the crucial importance of the hydrogen bonding interactions on the crystal stability. Moreover, the structure–activity relationship between the two crystalline phases has been well established and explained through the analysis of the crystal structure and the characterized macroscopic properties. The results of a series of stability experiments prove that the dihydrate has a higher stability than that of anhydrate of PDA-TDA. In particular, the transformation mechanism between the two crystal forms was found to be dominated by temperature and relative humidity, as dihydrate can transform into the anhydrate when held at 80 °C for 48 h, while the anhydrous forms can turn into dihydrate when the water content is more than 0.1 at 10 °C. Finally, nylon 514 products were polymerized using two different methods, MP and DSSP, employing dihydrate powders as the monomers. In contrast to the properties of the products obtained by MP, the nylons polymerized by DSSP show better performance in regards to their morphology, molecular weight, thermal stability, and water absorption. Considering the above performance characteristics, the nylon 514 product obtained in this work proved to be a novel bio-based long-chain nylon, with low water absorption, which exhibits potential for specific practical applications.

## Figures and Tables

**Table 1:** Detailed crystallographic information for the two forms of PDA–TDA.

	Anhydrate	Dihydrate
Empirical formula	C_14_H_24_O_4_, C_5_H_16_N_2_	C_14_H_24_O_4_, C_5_H_16_N_2_, 2H_2_O
Formula weight	360.53	396.53
Crystal system	triclinic	triclinic
Space group	*P*-1	*P*-1
*a* (Å)	5.7530(5)	8.4524(7)
*b* (Å)	9.5901(9)	8.5034(8)
*c* (Å)	21.1402(19)	16.9887(14)
*α* (deg)	96.590(2)	75.787(1)
*β* (deg)	94.309(2)	78.920(1)
*γ* (deg)	103.272(3)	86.523(2)
Volume (Å^3^)	1121.46(18)	1161.49(17)
*Z*	2	2
*D_calc_.*(g/cm^3^)	1.068	1.134
*μ* (mm^−1^)	0.074	0.083
*F* (000)	400	440
Observed data [I >2σ (I)]	1869	2554
*R*(int)	0.000	0.028
*R_1_* [I >2σ (I)]	0.2660	0.0549
*wR^2^*	0.5903	0.1604
GOF on F^2^	1.83	1.02
Diff density (e/Å^3^)	−0.56, 0.76	−0.18, 0.24
CCDC	2112957	2112969

**Table 2:** Comparison of the selected torsion angles (°) data for anhydrate and dihydrate.

Torsion Angles (°)	Anhydrate	Dihydrate
O1-C1-C2-C3	−133.8(10)	29.7(3)
O2-C1-C2-C3	54.9(13)	−153.1(2)
C1-C2-C3-C4	178.7(9)	68.4(3)
C11-C12-C13-C14	−177.9(11)	−173.2(2)
C12-C13-C14-O3	−57.2(14)	167.0(2)
C12-C13-C14-O4	131.5(11)	−12.2(3)
N1-C15-C16-C17	−52.9(15)	−56.6(3)
C15-C16-C17-C18	−66.9(13)	−177.19(19)
C16-C17-C18-C19	−179.5(10)	−177.42(19)
C17-C18-C19-N2	−58.5(16)	−177.15(18)

**Table 3:** Thermodynamic information for the two crystalline forms of PDA-TDA.

	Loss of Water Content	Theoretical Water Content	T_onset_/°C	∆H_eva_/J/g	^a^ E_latt_ (kJ/mol)
Anhydrate	/	/	106.7	272.0	−169.3
Dihydrate	9.31%	9.08%	117.9	295.8	−164.8

^a^: E_latt_ is the lattice energy, E_latt_ = E_bulk_/Z − E_A_ − E_B_ − E_C_ [78], where E_bulk_ represents the total energy of the cell unit. E_A_, E_B_, and E_C_ correspond to PDA, TDA, and the solvent molecules’ relaxed energy, respectively, and Z is the number of the smallest asymmetric units in the unit cell.

**Table 4:** Comparison of the molecular weight, water absorption, and thermodynamic properties for PA 514 obtained using MP and DSSP methods, respectively.

	*M*_n_ × 10^4^/Da	*M*_w_ × 10^4^/Da	PDI	[η]/dL/g	Water Absorption/%	T_m_/°C	T_c_/°C
PA514-MP	1.43	3.30	2.31	0.20	1.61	198.78	167.71
PA514-DSSP	1.85	3.53	1.91	0.37	0.06	203.89	168.42

## Data Availability

Data are contained within the article and Supplementary Materials.

## Supplementary Materials

The following supporting information can be downloaded at: https://www.mdpi.com/article/10.3390/polym16040480/s1, Table S1: Hydrogen bond data for the two crystal forms of PDA-TDA. Table S2: Binding energy and lattice energy calculations for the two forms of PDA-TDA. Figure S1: Comparation of the torsion angles in the smallest asymmetric units in the two crystalline forms of PDA-TDA: (a) anhydrate; (b) dihydrate. Figure S2: Comparation of the two forms of PDA-TDA in terms of packing coefficient and crystal density. Figure S3: Hirshfeld surface and its corresponding fingerprint plots of the anhydrate. Figure S4: Hirshfeld surface and its corresponding fingerprint plots of the dihydrate. Figure S5: C-H∙∙∙O contacts in the Hirshfeld surface shape index of the two crystalline forms of PDA-TDA: (a) anhydrous phase; (b) dihydrate. Figure S6: PXRD patterns of the anhydrous form (a) and dihydrate (b) under different relative humidity. Slurry experiments for the anhydrate and dihydrate mixtures under different water activities (water–ethanol solution) at 10–45 °C: (a) 10 °C; (b) 25 °C; (c) 35 °C; (d) 45 °C. Figure S8: FTIR analysis of the obtained PA514 products using MP and DSSP polymerization methods, respectively.

## References

[1] Winnacker M., Rieger B. (2016). Biobased Polyamides: Recent Advances in Basic and Applied Research. Macromol. Rapid Commun..

[2] Lee J.A., Ahn J.H., Kim I., Li S., Lee S.Y. (2020). Synthesis, Characterization, and Application of Fully Biobased and Biodegradable Nylon-4,4 and -5,4. ACS Sustain. Chem. Eng..

[3] Li M. (2019). Study on melting and polymorphic behavior of poly(decamethylene terephthalamide). J. Polym. Sci. Part B Polym. Phys..

[4] Deshmukh Y.S., Wilsens C.H.R.M., Verhoef R., Hansen M.R., Dudenko D., Graf R., Klop E.A., Rastogi S. (2016). Conformational and Structural Changes with Increasing Methylene Segment Length in Aromatic–Aliphatic Polyamides. Macromolecules.

[5] Kim Y.J., Yohana K.E., Lee H.-s., Kim J. (2012). Solid-State Polymerization of Semiaromatic Copolyamides of Nylon-4,T and Nylon-4,6: Composition Ratio Effect and Thermal Properties. Ind. Eng. Chem. Res..

[6] Anwar S., Hassanpour Amiri M., Jiang S., Abolhasani M.M., Rocha P.R.F., Asadi K. (2021). Piezoelectric Nylon-11 Fibers for Electronic Textiles, Energy Harvesting and Sensing. Adv. Funct. Mater..

[7] Yanaka A., Sakai W., Kinashi K., Tsutsumi N. (2020). Ferroelectric performance of nylons 6-12, 10-12, 11-12, and 12-12. RSC Adv..

[8] Li Y.-S., Schwarz G., Kricheldorf H.R. (2000). New polymers syntheses. 104. Synthesis of poly(ester-imide)s from nylon 6, nylon 11, or nylon 12. J. Polym. Sci. A Polym. Chem..

[9] Bhushan R.K., Sharma D. (2019). Green welding for various similar and dissimilar metals and alloys: Present status and future possibilities. Adv. Compos. Hybrid. Mater..

[10] Liu B., Hu G., Zhang J., Yan W. (2019). Non-isothermal crystallization, yellowing resistance and mechanical properties of heat-resistant nylon 10T/66/titania dioxide/glass fibre composites. RSC Adv..

[11] Wang T., Li H., Diao X., Lu X., Ma D., Ji N. (2023). Lignin to dispersants, adsorbents, flocculants and adhesives: A critical review on industrial applications of lignin. Ind. Crops Prod..

[12] Firdaus M., Meier M.A.R. (2013). Renewable polyamides and polyurethanes derived from limonene. Green Chem..

[13] Hashimoto K., Hashimoto N., Kamaya T., Yoshioka J., Okawa H. (2011). Synthesis and properties of bio-based polyurethanes bearing hydroxy groups derived from alditols. J. Polym. Sci. Part A Polym. Chem..

[14] Miri V., Persyn O., Lefebvre J.M., Seguela R. (2009). Effect of water absorption on the plastic deformation behavior of nylon 6. Eur. Polym. J..

[15] Li Z., Li S., Yang P., Fang X., Wen Q., Li M., Zhuang W., Wu J., Ying H. (2023). The effect of polymorphism on polymer properties: Crystal structure, stability and polymerization of the short-chain bio-based nylon 52 monomer 1,5-pentanediamine oxalate. IUCrJ.

[16] Osswald T.A., García-Rodríguez S. (2011). History of sustainable bio-based polymers. Green Chem..

[17] Yang P., Li X., Liu H., Li Z., Liu J., Zhuang W., Wu J., Ying H. (2019). Thermodynamics, crystal structure, and characterization of a bio-based nylon 54 monomer. CrystEngComm.

[18] Ogunniyi D.S. (2006). Castor oil: A vital industrial raw material. Bioresour. Technol..

[19] Hong S.H., Kim J.S., Lee S.Y., In Y.H., Choi S.S., Rih J.K., Kim C.H., Jeong H., Hur C.G., Kim J.J. (2004). The genome sequence of the capnophilic rumen bacterium Mannheimia succiniciproducens. Nat. Biotechnol..

[20] Xue Y., Zhao Y., Ji X., Yao J., Busk P.K., Lange L., Huang Y., Zhang S. (2020). Advances in bio-nylon 5X: Discovery of new lysine decarboxylases for the high-level production of cadaverine. Green Chem..

[21] Mi J., Liu S., Qi H., Huang J., Lan X., Zhang L. (2021). Cellular Engineering and Biocatalysis Strategies toward Sustainable Cadaverine Production: State of the Art and Perspectives. ACS Sustain. Chem. Eng..

[22] Li Y., Song X., Xu W., Duan X., Shi J., Li X. (2022). Preparation of biomass film from waste biomass energy corn stalk under carbon neutralization strategy. Mater. Today Commun..

[23] Ma Z., Qin S., Yao Y., Xin Z., Luan L., Zhang Y., Huang Y. (2023). Directed synthesis of nylon 5X key monomer cadaverine with alkaline metal modified Ru@FAU catalysts. Appl. Catal. A—Gen..

[24] Li Z., Wang K., Xue Y., Lin K., Ji X., Huang Y. (2023). Ionozymes for Efficient Synthesis of Cadaverine: Offering a Sustainable Way for Bio-nylon 5X Production. ACS Sustain. Chem. Eng..

[25] Bosch J. (2011). Modeling Techniques for Measuring Galaxy Properties in Multi-Epoch Surveys. J. Bus. Strategy.

[26] Burke D.J., Kawauchi T., Kade M.J., Leibfarth F.A., McDearmon B., Wolffs M., Kierstead P.H., Moon B., Hawker C.J. (2012). Ketene-Based Route to rigid Cyclobutanediol Monomers for the Replacement of BPA in High Performance Polyesters. ACS Macro Lett..

[27] Fleming M.E., Swift J.A. (2023). Enhancement of Hydrate Stability through Substitutional Defects. Cryst. Growth Des..

[28] Bui M., Nagapudi K., Chakravarty P. (2022). Determination of BET Specific Surface Area of Hydrate-Anhydrate Systems Susceptible to Phase Transformation Using Inverse Gas Chromatography. AAPS PharmSciTech.

[29] Cui P., Yin Q., Guo Y., Gong J. (2012). Polymorphic Crystallization and Transformation of Candesartan Cilexetil. Ind. Eng. Chem. Res..

[30] Brittain H.G. (1999). Polymorphism in Pharmaceutical Solids.

[31] Fujii K., Aoki M., Uekusa H. (2013). Solid-State Hydration/Dehydration of ErythromycinA Investigated by ab Initio Powder X-ray Diffraction Analysis:Stoichiometric and Nonstoichiometric Dehydrated Hydrate. Cryst. Growth Des..

[32] Liu Z., Yin Q., Zhang X., Gong J., Xie C. (2014). Characterization and Structure Analysis of Cefodizime Sodium Solvates Crystallized from Water and Ethanol Binary Solvent Mixtures. Ind. Eng. Chem. Res..

[33] SeethaLekshmi S., Kiran M.S.R.N., Ramamurty U., Varughese S. (2020). Phase Transitions and Anisotropic Mechanical Response in a Water-rich Trisaccharide Crystal. Cryst. Growth Des..

[34] Liu F., Hooks D.E., Li N., Mara N.A., Swift J.A. (2018). Mechanical Properties of Anhydrous and Hydrated Uric Acid Crystals. Chem. Mater..

[35] Noguerol A.T., Marta Igual M., Pagán M.J. (2022). Developing psyllium fibre gel-based foods: Physicochemical, nutritional, optical and mechanical properties. Food Hydrocoll..

[36] Zhu B., Zhang Q., Ren G., Mei X. (2017). Solid-State Characterization and Insight into Transformations and Stability of Apatinib Mesylate Solvates. Cryst. Growth Des..

[37] Zhu B., Wang J.-R., Zhang Q., Li M., Guo C., Ren G., Mei X. (2018). Stable Cocrystals and Salts of the Antineoplastic Drug Apatinib with Improved Solubility in Aqueous Solution. Cryst. Growth Des..

[38] Kocian Š., Štejfa V., Rohlíček J., Červinka C. (2023). Calorimetric and Crystallographic Phase-Behavior Study of Selected 1-Butylpyridinium Ionic Liquids. Cryst. Growth Des..

[39] Du W., Yin Q., Hao H., Bao Y., Zhang X., Huang J., Li X., Xie C., Gong J. (2014). Solution-Mediated Polymorphic Transformation of Prasugrel Hydrochloride from Form II to Form I. Ind. Eng. Chem. Res..

[40] Steendam R.R.E., Khandavilli U.B.R., Keshavarz L., Frawley P.J. (2019). Solution versus Crystal Hydration: The Case of γ-Amino Acid Pregabalin. Cryst. Growth Des..

[41] Boeckmann J., Näther C. (2010). Solid-state transformation of [Co(NCS)2(pyridine)4] into [Co(NCS)2(pyridine)2]n: From Curie-Weiss paramagnetism to single chain magnetic behaviour. Daltont.

[42] Liu F., Sommer F., Bos C., Mittemeijer E.J. (2007). Analysis of solid state phase transformation kinetics: Models and recipes. Metall. Rev..

[43] Zou F., Chen Q., Yang P., Zhou J., Wu J., Zhuang W., Ying H. (2017). Solution-Mediated Polymorphic Transformation: From Amorphous to Crystals of Disodium Guanosine 5′-Monophosphate in Ethanol. Ind. Eng. Chem. Res..

[44] Maher A., Seaton C.C., Hudson S., Croker D.M., Rasmuson Å.C., Hodnett B.K. (2012). Investigation of the Solid-State Polymorphic Transformations of Piracetam. Cryst. Growth Des..

[45] Chee-wei Jennifer L., Ronald W.R. (2006). Solubilities of and Transformations between the Anhydrous and Hydrated Forms of L-Serine in Water−Methanol Solutions. Cryst. Growth Des..

[46] Maruyama S., Ooshima H., Kato J. (1999). Crystal structures and solvent-mediated transformation of Taltireline polymorphs. Chem. Eng. J..

[47] Bērziņš A., Trimdale-Deksne A., Belyakov S., ter Horst J.H. (2023). Switching Nitrofurantoin Polymorphic Outcome in Solvent-Mediated Phase Transformation and Crystallization Using Solvent and Additives. Cryst. Growth Des..

[48] Bruker (2012). SAINT and SMART-1000.

[49] Sheldrick G.M. (2015). SHELXT-Integrated space-group and crystal-structure determination. Acta Cryst..

[50] Usón I., Sheldrick G.M. (2018). An introduction to experimental phasing of macromolecules illustrated by SHELX: New autotracing features. Acta Cryst..

[51] Atwood J.L., Barbour L.J. (2003). Molecular Graphics:  From Science to Art. Cryst. Growth Des..

[52] Spek A.L. (2003). Single-crystal structure validation with the program PLATON. J. Appl. Cryst..

[53] Gavezzotti A., Filippini G. (1994). Geometry of the Intermolecular X-H.cntdot..cntdot..cntdot.Y (X, Y = N, O) Hydrogen Bond and the Calibration of Empirical Hydrogen-Bond Potentials. J. Phys.Chem..

[54] Dolomanov O.V., Bourhis L.J., Gildea R.J., Howard J.A.K., Puschmann H. (2010). OLEX2: A complete structure solution, refinement and analysis program. J. Appl. Cryst..

[55] Hirshfeld F.L. (1977). Bonded-atom fragments for describing molecular charge densities. Theor. Chim. Acta.

[56] Spackman M.A., Jayatilaka D. (2009). Hirshfeld surface analysis. CrystEngComm.

[57] Spackman M.A. (2013). Molecules in crystals. Phys. Scr..

[58] Spackman M.A., McKinnon J.J. (2002). Fingerprinting intermolecular interactions in molecular crystals. CrystEngComm.

[59] Turner M.J., McKinnon J.J., Wolff S.K., Grimwood D.J., Spackman P.R., Jayatilaka D., Spackman M.A. (2017). CrystalExplorer17.

[60] Grimme S., Ehrlich S., Goerigk L. (2011). Effect of the damping function in dispersion corrected density functional theory. J. Comput. Chem..

[61] Grimme S. (2011). Density functional theory with London dispersion corrections. WIREs Comput. Mol. Sci..

[62] Lu T., Chen Q. (2021). Interaction Region Indicator: A Simple Real Space Function Clearly Revealing Both Chemical Bonds and Weak Interactions. ChemRxiv.

[63] Lee C., Yang W., Parr R.G. (1988). Development of the Colle-Salvetti correlation-energy formula into a functional of the electron density. Phys. Rev. B.

[64] Becke A.D. (1993). Density-functional thermochemistry. III. The role of exact exchange. J. Chem. Phys..

[65] Frisch M.J., Trucks G.W., Schlegel H.B., Scuseria G.E., Robb M.A., Cheeseman J.R., Scalmani G., Barone V., Mennucci B., Petersson G.A. (2009). Gaussian 09, Revision A.02.

[66] Lu T., Chen F. (2012). Multiwfn: A multifunctional wavefunction analyzer. J. Comput. Chem..

[67] Kalra A., Tishmack P., Lubach J.W., Munson E.J., Taylor L.S., Byrn S.R., Li T. (2017). Impact of Supramolecular Aggregation on the Crystallization Kinetics of Organic Compounds from the Supercooled Liquid State. Mol. Pharm..

[68] Humphrey W., Dalke A., Schulten K. (1996). VMD: Visual molecular dynamics. J. Mol. Graph..

[69] Li M., Wang M., Liu Y., Ouyang R., Liu M., Han D., Gong J. (2021). Co-amorphization Story of Furosemide-Amino Acid Systems: Protonation and Aromatic Stacking Insights for Promoting Compatibility and Stability. Cryst. Growth Des..

[70] Singh M.K., Banerjee A. (2013). Role of Solvent and External Growth Environments to Determine Growth Morphology of Molecular Crystals. Cryst. Growth Des..

[71] Yang P., Lin C., Zhuang W., Wen Q., Zou F., Zhou J., Wu J., Ying H. (2016). Insight into a direct solid–solid transformation: A potential approach for the removal of residual solvents. CrystEngComm.

[72] Wei X.-W., Huang G., Wang J., Meng X., Zhou Q., Ye H.-M. (2020). Tailoring Crystallization of Random Terpolyester: Combination of Isodimorphism and Isomorphism. Macromolecules.

[73] Jamieson C.S., Mebel A.M., Kaiser R.I. (2006). Understanding the Kinetics and Dynamics of Radiation-induced Reaction Pathways in Carbon Monoxide Ice at 10 K. Astrophys. J. Suppl. Ser..

[74] Boussia A.C., Vouyiouka S.N., Porfiris A.D., Papaspyrides C.D. (2010). Long-Aliphatic-Segment Polyamides: Salt Preparation and Subsequent Anhydrous Polymerization. Macromol. Mater. Eng..

[75] Bernstein J. (2007). Polymorphism in Molecular Crystals.

[76] Zhang X., Zhou L., Wang C., Li Y., Wu Y., Zhang M., Yin Q. (2017). Insight into the role of hydrogen bonding playing in the molecular self-assembly process of sulfamethazine solvates. Cryst. Growth Des..

[77] Papaspyrides C.D., Porfyris A.D., Vouyiouka S., Rulkens R., Grolman E., Poel G.V. (2016). Solid state polymerization in a micro-reactor: The case of poly(tetramethylene terephthalamide). J. Appl. Polym. Sci..

[78] Jia L., Zhang S., Yang W., Bao Y., Hou B., Zhou L., Yin Q. (2021). Insights into Intermolecular Interactions of Spironolactone Solvates. Cryst. Growth Des..

