# Supplementary material for: Long-Chain Bio-Based Nylon 514 Salt: Crystal Structure, Phase Transformation, and Polymerization
Source: Polymers (Basel). 2024 Feb 8;16(4):480. doi: 10.3390/polym16040480 (PMC10892662; doi:10.3390/polym16040480)
Supplement: Supplementary file 1 [file polymers-16-00480-s001.zip › polymers-2774640-supplementary.pdf]

Supplementary Materials

Table S1 Hydrogen bond data for the two crystal forms of PDA-TDA.

**Anhydrate**				
D-H···A	D(D-H) / Å	d(H···A) / Å	d(D···A)/ Å	D-H···A
N1-H1A···O2	0.8900	1.9200	2.716(12)	148.00
N1-H1B···O4	0.8900	2.0200	2.789(11)	144.00
N1-H1C···O1	0.8900	2.0200	2.841(11)	152.00
N2-H2A···O1	0.8900	2.0400	2.845(10)	149.00
N2-H2B···O4	0.8900	1.9700	2.818(11)	160.00
N2-H2C···O3	0.8900	1.9100	2.757(12)	157.00
**Dihydrate**				
D-H···A	D(D-H) / Å	d(H···A) / Å	d(D···A)/ Å	D-H···A
N1-H1A···O3	0.8900	1.9000	2.775(2)	167.00
N1-H1B···O1	0.8900	1.8900	2.773(2)	174.00
N1-H1C···O6	0.8900	1.8600	2.721(3)	163.00
N2-H2A···O2	0.8900	1.9200	2.808(2)	172.00
N2-H2B···O3	0.8900	1.9100	2.791(2)	169.00
N2-H2C···O5	0.8900	1.9600	2.819(2)	162.00
O5-H5E···O1	0.8500	1.9100	2.747(2)	167.00
O5-H5F···O4	0.8500	1.9100	2.747(2)	167.00
O6-H6E···O2	0.8500	1.9500	2.798(2)	179.00
O6-H6F···O4	0.8500	1.8400	2.692(2)	180.00

Figure S1 Comparation of the torsion angles in the smallest asymmetric units in the two crystalline forms of PDA-TDA: (a) anhydrate; (b) dihydrate.

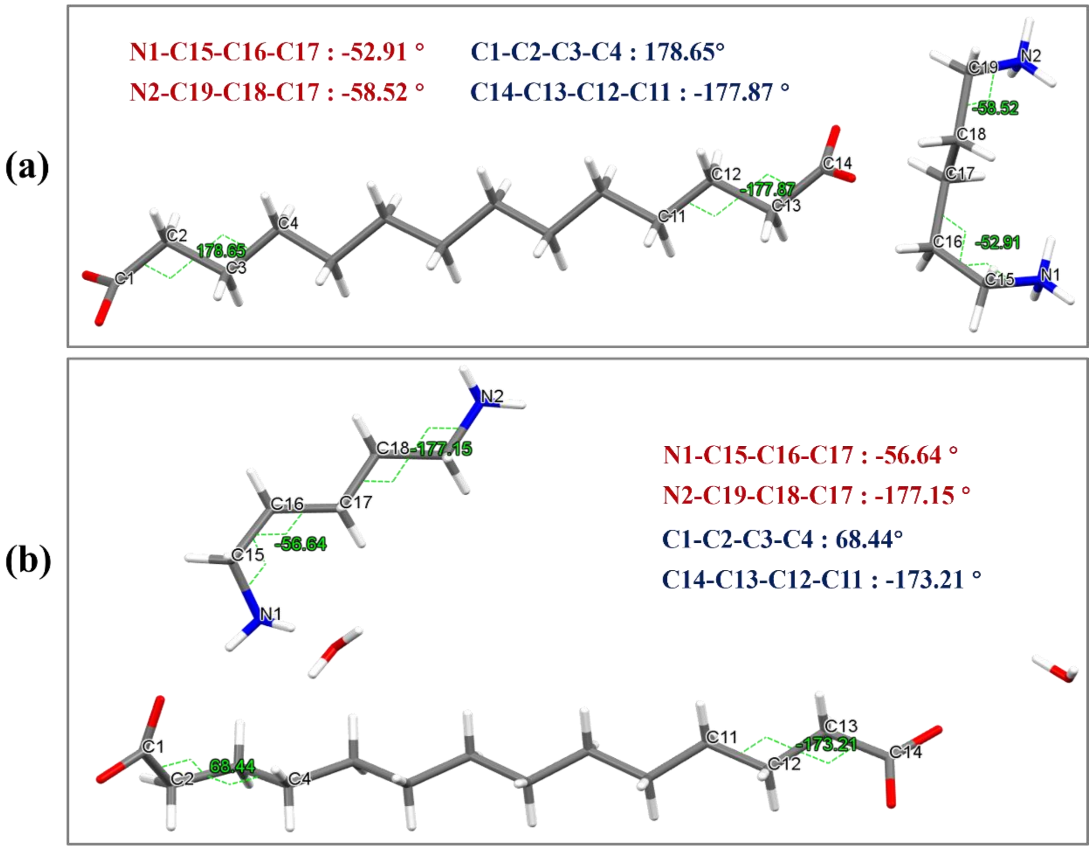

Figure S2 Comparation of the two forms of PDA-TDA in terms of packing coefficient and crystal density.

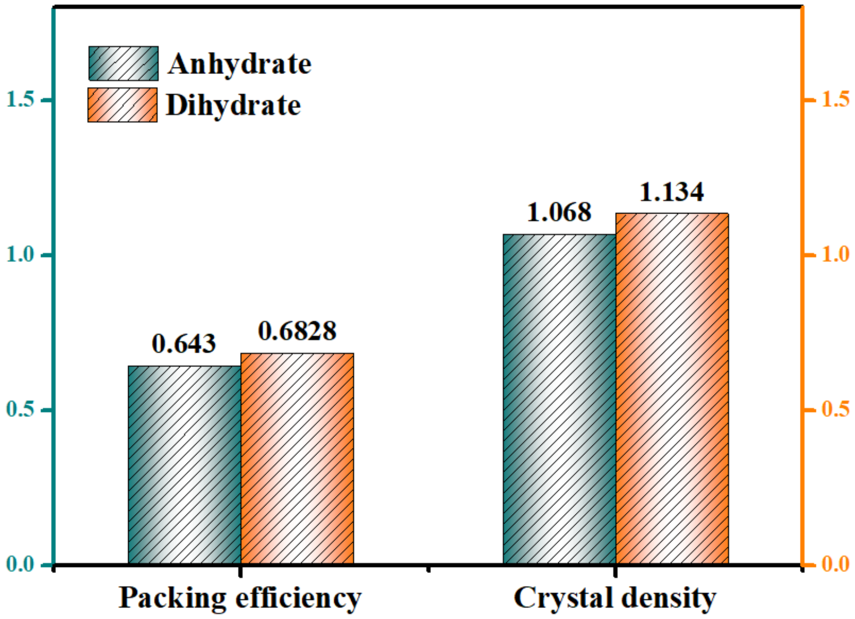

Figure S3 Hirshfeld surface and its corresponding fingerprint plots of the anhydrate.

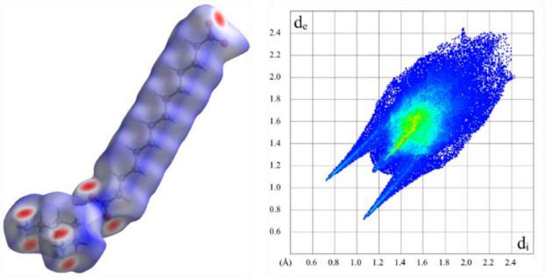

(a) All···All

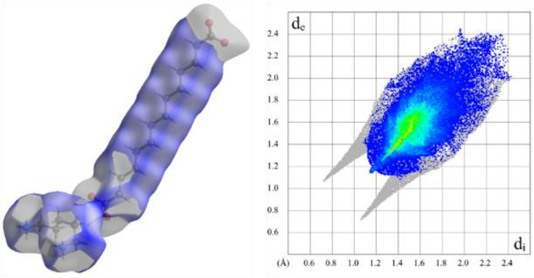

(b) H···H (70.7%)

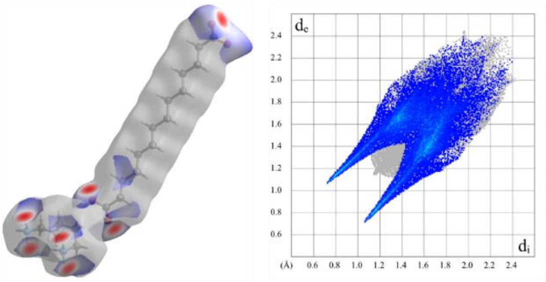

(c) H···O/O···H (28.3%)

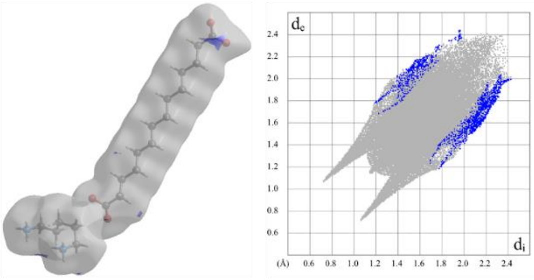

(d) H···C/C···H (1.0%)

Figure S4 Hirshfeld surface and its corresponding fingerprint plots of the dihydrate.

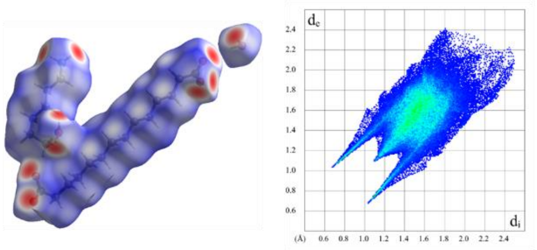

(a) All···All

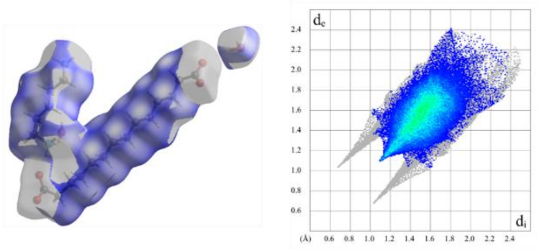

(b) H···H (69.8%)

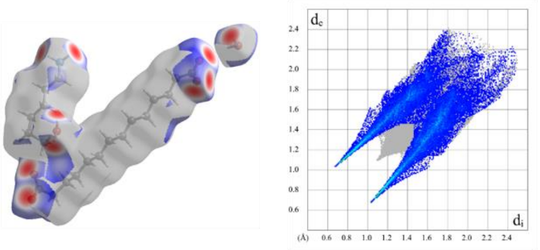

(c) H···O/O···H (29%)

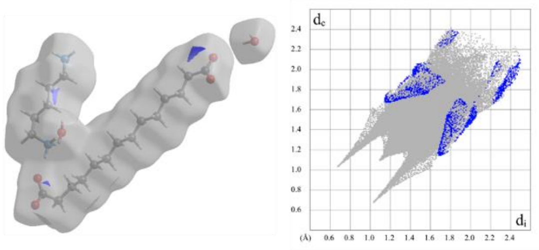

(d) H···C/C···H (1.2%)

Figure S5 C-H···O contacts in the Hirshfeld surface shape index of the two crystalline forms of PDA-TDA: (a) anhydrous phase; (b) dihydrate.

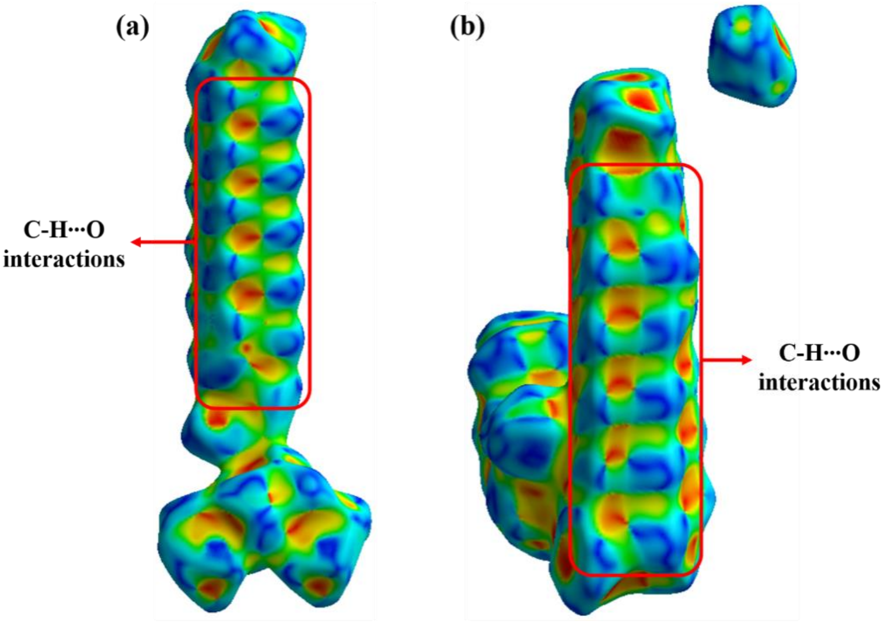

Table S2 Binding energy and lattice energy calculations for the two forms of PDA-TDA.

	Anhydrate	Dihydrate
E_{bind} (kcal/mol)	-12554.45	-13515.45
E_{latt} (kJ/mol)	-169.30	-164.78

Figure S6 PXRD patterns of the anhydrous form (a) and dihydrate (b) under different relative humidity.

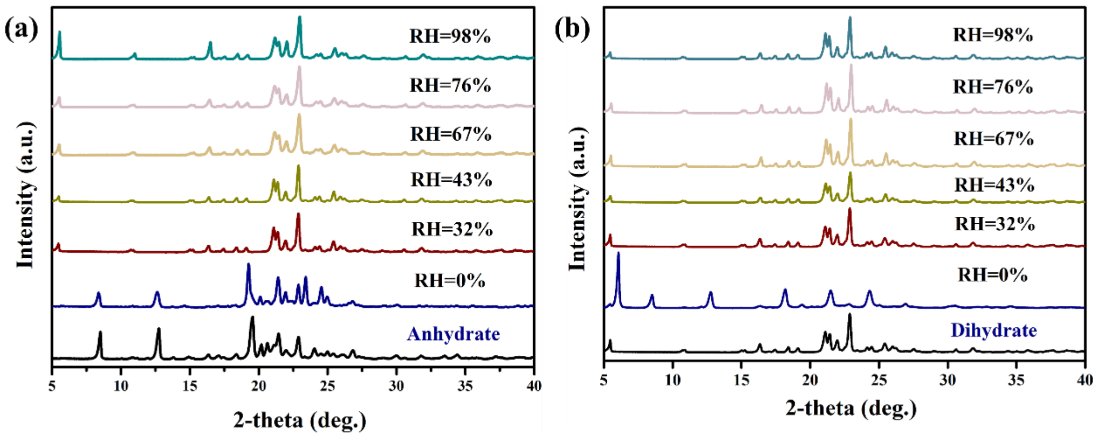

Figure S7 Slurry experiments for the anhydrate and dihydrate mixtures under different water activities (water – ethanol solution) at 10 °C–45 °C: (a) 10 °C; (b) 25 °C; (c) 35 °C; (d) 45 °C.

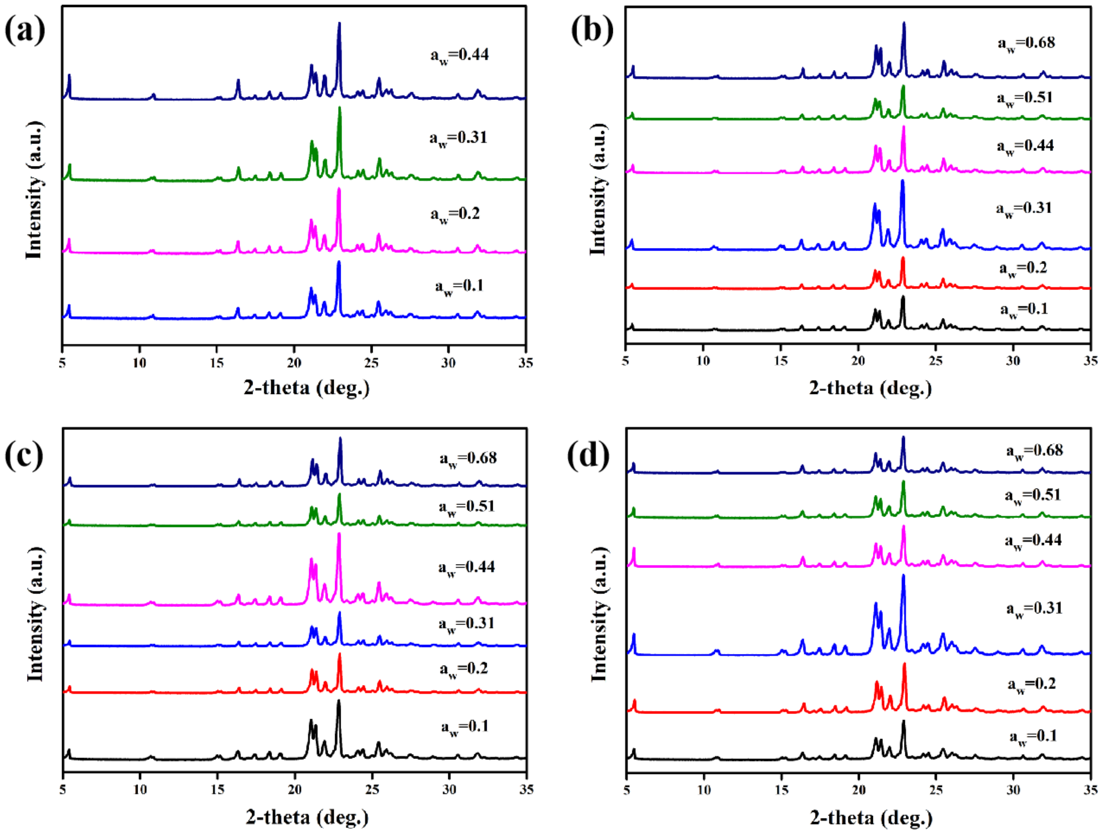

Figure S8 FTIR analysis of the obtained PA514 products using MP and DSSP polymerization methods, respectively.

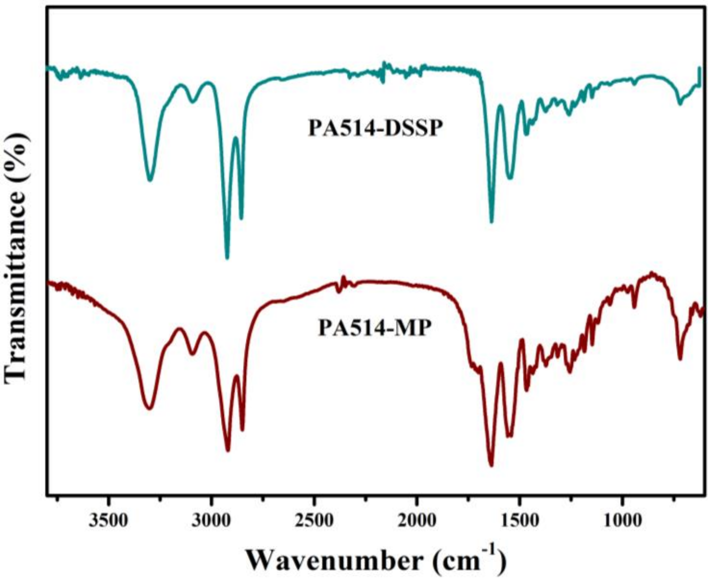

1. Preparation of the two crystal forms of PDA-TDA.

Anhydrate: Weighed approximately 5 ± 0.1 g of PDA and 12.64 ± 0.1 g of TDA crystalline powder, dissolved the mixture in 60 ml of methanol. After dissolved completely, transferred the PDA-TDA methanol solution into a 500-ml double-jacketed crystallizer with a 180 r/min of stirring rate. Following, pump the 2-propanol solvent slowly into the crystallizer using a peristaltic pump at 2 ml/min. With the adding of anti-solvent, the precipitate was observed after about 30 min. Keep stirring for 2 hours, then the anhydrous crystals can be obtained.

Dihydrate. A total of 5 ± 0.1 g of the PDA and 12.64 ± 0.1 g of TDA powder was dissolved in 50 ml of water and then poured the solution into a 500-ml double-jacketed crystallizer, agitated at 180 r/min until the mixed solution became transparent at ambient temperature. Then pump the 2-propanol solvent slowly into the PDA-TDA aqueous solution at a 2 ml/min constant rate. Flake-like crystals of dihydrate gradually appeared with the addition of 2-propanol for about 45 min. After 2 h, filtered the dihydrate crystalline product and then dried at 60 °C to be tested.

2. Preparation of single crystals of two crystal forms.

Suitable single crystals of anhydrate and dihydrate were obtained by cooling crystallization. An excess of powder of the dihydrate was dissolved in 10 ml pure water, stirred thoroughly and the solid completely dissolved at 60 °C. The solution was filtered with a 0.45 μm nylon filter, then transferred to a 10 ml glass vial. Cool down slowly at a rate of 1 °C/h until room temperature, then the lamellar dihydrate single crystal precipitated out after about 2 hours. The extraction of anhydrate single crystals is almost the same as the dihydrate, except that the solvent is DMF. Through the same steps of dissolution, filtration, and cooling as mentioned above, the crystal of anhydrate can be obtained after several days.

3. Polymerization Experiments.

Melting polymerization (MP). Firstly, the PDA-TDA dihydrate salt was weighted and dissolved in deionized water (m_{salt}:m_{water}=1:2) and transferred to the autoclave (AC100ml, Beijing Century Senlong) after completely stirring. Secondly, the

mixture was purged with nitrogen three times in the autoclave and heated to 130 °C for 0.5 h at a pressure of 0.26 MPa. Next, the temperature was raised up to 180 ℃ and the mixture was held for 1 h, under a pressure of 1.08 MPa. These two steps inhibit the escape of the volatile diamine, thus forcing it to react. Thirdly, the pressure was slowly reduced to atmospheric pressure within 1.5 h in order to remove the by-product and increase the molecular weight of the nylon product. Finally, the product was held under 0.095 MPa negative pressure for 4 h, which was applied to further increase the molecular weight of the formed pre-polymer. After the experiment, the synthesized polymer was removed from the autoclave.

Direct solid-state polymerization (DSSP). A total of 15 g PDA-TDA monomer powders were put into a 100 ml autoclave, using high-purity nitrogen gas, which was replaced for three times. The DSSP procedures are as follows: (1) The autoclave was heated up to 120 ℃, and the pressure inside the autoclave was kept at 0.5 MPa for 1 h. (2) The temperature was raised to 170 ℃, with a heating rate of 0.2 ℃/min, and the pressure was maintained at 0.8 MPa for 1 h. (3) The pressure inside the autoclave was slowly reduced to the atmospheric pressure within 1.5 h, and this pressure was then maintained for 4 h; this step is helpful for increasing the molecular weight of nylons. (4) Lastly, the final product was removed from the bottom of the autoclave after the conclusion of the experiment.

4. Characterization of bio-nylon 514.

4.1. Molecular weight analysis. Using hexafluoroisopropyl alcohol (HFIP) as the solvent, gel permeation chromatography (GPC) was used to determine the mass average molecular weight (M_w), the number average molecular weight (M_n), and the polydispersity index (PDI), taking PMMA as the standard sample. A total of 3 mg of nylon 514 powder was weighed, at room temperature, and prepared into a solution with a concentration of $1x10^{-3}$ g/ml; each sampling volume was about 20-25 μl, with an injection rate of 1 ml/min.

4.2. Intrinsic viscosity measurement. A total of 0.125 g of nylon 514 polymer was accurately weighed and dissolved in 25 ml of concentrated sulfuric acid to prepare a

nylon 514 solution with a concentration of 0.5 gd/L. The discharge time of the solution was measured and recorded in a water bath at 25 °C using a viscosimeter. The average time was captured three times, and the interval time was within 0.2 s. The relative and incremental viscosities of the nylon 514 product were calculated by comparing the outflow time of the pure concentrated sulfuric acid solution with that of the nylon 514 solution. The Solomon–Ciuta equation (Equation 1) was used to determine the single point intrinsic viscosities, and the Mark–Houwink equation (Equation 2) was used to estimate the mean molecular weight M_n, where, η_{sp} is the increased viscosity, η_{rel} is the relative viscosity, and C is the concentration. The Mark–Houwink constant K = 11.5×10^{-4} dL/g and α = 0.67 are used to estimate the viscosity of bio-based nylon 514 in this work.

$$[\eta] = \frac{\sqrt{2\times[\eta_{sp} - \ln{(\eta_{rel})}]}}{C} \quad (1)$$

$$[\eta] = K\left(\frac{dL}{g}\right) * M_n^{\alpha} \quad (2)$$

4.3. Water absorption analysis. A tablet press was chosen to make the sheet of nylon 514. The test condition was 250 °C, the sheet was pressed under 10 MPa for 5 min, and the obtained polymer thickness was about 5-10 mm. The obtained nylon 514 polymer sheets were weighed, and the weight and quality were recorded. The polymer sheets were soaked in an aqueous solution for 24 hours, after which the sheets were removed and weighed again. Water absorption was calculated using the difference between the polymer mass before and after soaking.

4.4. Thermodynamic analysis. The thermodynamic properties of the nylon 514 polymer were obtained using DSC. Using N_2 as the protective carrier gas, a 3 mg sample was accurately weighed and then placed it in an alumina crucible. The measuring temperature range was from 30 to 350 °C, and both the heating and cooling rates were constant at 10 °C/min.
